# Antioxidant for Neurological Diseases and Neurotrauma and Bioengineering Approaches

**DOI:** 10.3390/antiox11010072

**Published:** 2021-12-29

**Authors:** Nasera Rizwana, Vipul Agarwal, Manasa Nune

**Affiliations:** 1 Manipal Institute of Regenerative Medicine (MIRM), Bengaluru, Manipal Academy of Higher Education (MAHE), Manipal 576104, India; nasera.rizwana@learner.manipal.edu; 2 Cluster for Advanced Macromolecular Design (CAMD), School of Chemical Engineering, University of New South Wales, Sydney, NSW 2052, Australia

**Keywords:** antioxidants, oxidative stress, neurotrauma, neuroregeneration, bioengineering approaches

## Abstract

Antioxidants are a class of molecules with an innate affinity to neutralize reactive oxygen species (ROS), which are known to cause oxidative stress. Oxidative stress has been associated with a wide range of diseases mediated by physiological damage to the cells. ROS play both beneficial and detrimental roles in human physiology depending on their overall concentration. ROS are an inevitable byproduct of the normal functioning of cells, which are produced as a result of the mitochondrial respiration process. Since the establishment of the detrimental effect of oxidative stress in neurological disorders and neurotrauma, there has been growing interest in exploring antioxidants to rescue remaining or surviving cells and reverse the neurological damage. In this review, we present the survey of different antioxidants studied in neurological applications including neurotrauma. We also delve into bioengineering approaches developed to deliver antioxidants to improve their cellular uptake in neurological applications.

## 1. Introduction

Oxidative stress induced by reactive oxygen species (ROS) is inevitably produced from normal cellular metabolism through mitochondrial respiration and a family of membrane-bound NADPH oxidases (NOXs). ROS are broadly divided into free radicals (molecules with unpaired electrons) and non-radical species. Physiologically, three main ROS types are superoxide anion (O_2_^−^), hydroxyl radical (OH^·^), and hydrogen peroxide (H_2_O_2_) [1]. ROS play an important role in both physiological cell functioning at low to moderate concentrations by oxidizing different collocated proteins to activate multiple biochemical pathways associated with cell viability, proliferation, differentiation and metabolic adaptation [2]. However, at high concentrations, ROS are toxic and cause oxidation-induced damage to the pivotal cell components including lipids, proteins and DNA leading to cell cycle arrest and cell death [3,4,5,6,7]. There is growing evidence showcasing that the excess oxidative stress cause different pathological diseases including cancer, atherosclerosis, neurological disorders, cardiovascular stress (hypertension, ischemia, reperfusion), diabetes mellitus, chronic inflammation, acute respiratory distress syndrome, idiopathic pulmonary fibrosis, chronic obstructive pulmonary disease, and asthma [8,9,10]. For example, atherosclerosis is caused by ROS-mediated oxidation of the lipids in low-density lipoprotein (LDL) called lipid peroxidation [11]. In cancer, ROS can promote cancer by introducing conducive genetic mutations by oxidizing specific intracellular chemical moieties and activating biochemical pathways that promote growth and neoplastic transformation [12]. Cancer cells survive high ROS concentrations by preserving the intrinsic concentrations of reduced glutathione and thioredoxin, which allows cells to activate proximal signaling pathways necessary for their neoplastic transformation and simultaneous repair of any damage caused by ROS to inhibit cell death.

Oxidative stress is central to neuropathologies including neurotrauma and neurodegenerative diseases. In neurotrauma, overproduction of ROS, in addition to Ca^+2^ imbalance, causes continued damage to the central nervous system known as secondary degeneration [13]. This overproduction of ROS causes a deficit in myelin which provides the structural basis for neuronal signal transmission. Damage to myelin causes neuronal damage, which can be exacerbated by the loss of oligodendrocytes (through overexposure to ROS). Oligodendrocytes are more vulnerable to ROS due to their higher intracellular iron levels and lower concentrations of antioxidants (such as glutathione and MnSOD) compared to glial cells [13,14]. ROS was implicated to induce the pathogenesis of neurodegenerative diseases (such as Alzheimer’s and Parkinson’s disease). Although the etiology of neurodegenerative diseases is not entirely understood, however, there is growing support for the role of ROS in augmenting disease progression. It is believed that in AD and PD, commonly found pathologies of aggregation of misfolded proteins can trigger an inflammatory response, which in turn induces ROS production [15,16]. Furthermore, ROS production from mitochondrial dysfunction in neurodegenerative diseases further exacerbates inflammation and dysregulates downstream redox signaling pathways, which were implicated in continued cell death [17]. Given the mixed reports on the efficacy of exogenous therapeutic antioxidants on ROS production in neurodegenerative diseases, there are multiple avenues of investigation currently undergoing to decipher the exact mechanism of ROS on the pathology of neurodegenerative diseases [17,18].

In the normal functioning of cells, ROS generated from mitochondrial functioning gets neutralized by the presence of endogeneous antioxidants. It is to be noted that the amount of endogenous antioxidants is highly cell type-dependent. Quantification of the endogenous antioxidant amount is not trivial despite the availability of assays such as (2,2-diphenyl-1-picrylhydrazyl) DPPH [19,20,21]. Despite this, methods are available to quantify the amount of generated ROS both in vitro and in vivo [22,23,24,25]. Due to the variability in the amount of endogenous antioxidants in different cell types, there has been a drive to explore exogenous antioxidants to supplement the supply and mitigate generating ROS. Exogenous antioxidants can be either natural or synthetic in origin. Natural antioxidants are of two types: enzyme-based and non-enzymatic. Enzymatic antioxidants include catalase [26], glutathione peroxidase [27], and superoxide dismutase [28]. Non-enzymatic antioxidant includes vitamin E [29], ascorbic acid [30], lipoic acid [31], polyphenols [32], and carotenoids [33]. Synthetic antioxidants include curcumin [34], chitosan (CS) [35], carbon nanotubes [36], polyurethane [37], nanoceria [38], melatonin [39], fullerene [40], tannic acid [41], melanin [42], and nitrones [43,44,45,46].

In general, there has been (pre)clinical, epidemiological, bench research evidence indicating that antioxidants can counteract and neutralize ROS and associated oxidative stress [9,47,48,49,50]. The mechanism of antioxidants includes (i) physically restricting ROS generation, (ii) functioning as chemical quenchers neutralizing ROS, (iii) acting as a catalyst (enzymes) to neutralize ROS, (iv) binding to metal ions to restrict ROS generation, (v) scavenging ROS by breaking radical chain reactions [8]. However, several studies have also revealed that excessive dietary intake of antioxidants exhibits little to no therapeutic effect [51,52,53]. Despite these conflicting reports, there remains a huge appetite for the clinical application of antioxidants for a wide range of modalities. This has motivated the use of antioxidants in the food industry as a preservative or/and for fortification to increase their intake [50]. There was anecdotal (from traditional medicine) and some clinical, epidemiological evidence correlating the positive effect of natural (plant-based) antioxidants [54,55,56]. However, the physiological effects of over-the-counter antioxidant supplements are not entirely clear. It could be due to (i) slow uptake of exogenous supplemented antioxidants, (ii) supplemented antioxidants may only target intracellular environment with low to no access to endogenously generated (mitochondrial- and NOX-derived) ROS [2]. One of the approaches to address these limitations could be slow release of dietary antioxidants and use of mitochondrial-targeted antioxidants. To achieve slow release of dietary antioxidants, there has been growing interest in the development of delivery methodologies, which was motivated by the advancements made in the field of biomedical engineering and nanotechnology-mediated drug delivery. Simultaneously, new antioxidants were extracted and studied from different natural sources with potentially higher internalization efficiency to access and neutralize intracellular ROS pools. There are continued efforts to understand the mechanism of action of all different types of antioxidants. We direct the attention of readers to a recent review from Nimse and Pal [57] on the mechanism of natural antioxidants. In this review, we focus on outlining the effect of antioxidants in neurological diseases and neurotrauma, followed by outlining different antioxidants studies in these modalities, and associated bioengineering approaches for antioxidant delivery for neuronal regeneration applications in both central and peripheral nervous systems. To this end, we concentrated on the literature published in the last 15 years (2005 onwards). It is envisaged that this review will inform and inspire the readers towards further research in the field of antioxidants and exploring their clinical translation especially towards neurological diseases and disorders.

## 2. Role of Oxidative Stress in Central Nervous System

Groups of free radicals such as superoxide anion, hydroxyl free radical, hydrogen peroxide, hypochlorous acid, singlet oxygen do not come under the exact definition of free radicals and are collectively called ROS [58]. At lower ROS concentrations, they behave as signaling molecules that are involved in cell proliferation and apoptosis and are considered essential for neuronal development and function in the central nervous system (CNS) [59,60]. At higher concentrations, they contribute against pathogens affecting the body. However, when their accumulation increases, they cause cell damage and changes in the DNA causing cell death. ROS are produced as a by-product of oxidation that propagates oxidative chain reactions. When this chain reaction occurs without any known cause and at mild environmental conditions, it is known as autoxidation. When peroxidases are formed as byproducts, it is known as peroxidation. During the oxidative chain reaction, the imbalance between the production and degradation of ROS causes its accumulation leading to cellular damage and cell death [61]. This imbalance is referred to as oxidative stress.

Oxidative stress negatively impacts CNS functions which are mainly because of the damage that occurs to the brain. Increased vulnerability of the brain to oxidative stress is due to its high oxygen demand, presence of high content of polyunsaturated fatty acids (PUFA) in the nerve cell membrane, and accumulation of transition metals [62]. Firstly, the brain is known to use around 20% of the energy of the whole human body due to its high metabolic rate. This energy is produced by mitochondria in the brain cells. Among the brain cells, neurons use about 70–80% of the energy, and the remaining is utilized by the glial cells [63]. Mitochondria produces about 80% of ROS in brain cells which helps in a normal cellular process. Due to leakage of electrons at four complexes (I–IV), ROS levels increase beyond the threshold leading to stress at the molecular level. This increase in ROS level causes DNA oxidation, lipid peroxidation, and protein oxidation. Secondly, the brain also contains a large amount of PUFA majorly docosahexaenoic acid, which increases lipid peroxidation. Lipid peroxidation involves free radical-dependent degradation of lipids. Free radical attack on PUFA present on the neural cell membrane releases highly active aldehydes, which in turn (a) increases the rigidity of the membrane, (b) hinders sodium pump activity, (c) alters membrane permeability, and (d) causes cell damage [64]. Thirdly, the major cause of oxidative stress in CNS is the accumulation of transition metals. Transition metals such as iron, copper, and zinc are required in biological reactions including cell proliferation and differentiation, DNA synthesis, and neurotransmitter synthesis. In addition, iron and copper are known catalysts for ROS production in cells [42]. Due to the increased levels of these metals, ROS production increases, thereby increasing lipid peroxidation leading to oxidative stress and cell damage.

## 3. Role of Oxidative Stress in Neurodegenerative Diseases

In Parkinson’s disease (PD) genesis, activation of glial cells and enzymes such as reduced nicotinamide adenine dinucleotide phosphate oxidase, inducible nitric oxide synthase, and astrocytic myeloperoxidase, as well as inflammatory factors, such as tumor necrosis factor α and cyclooxygenase-2 play an important role [65]. The progression of PD is mainly attributed to free radical-induced oxidative stress which causes nucleic acid instability, increased mitochondrial DNA mutation, and protein homeostasis disruption [66]. The free radical-mediated oxidative stress damage the dopaminergic neurons in substantia nigra through lipid peroxidation, protein peroxidation, DNA oxidation, alteration in iron content, and monoamine oxidase activation.

**Figure 1:**
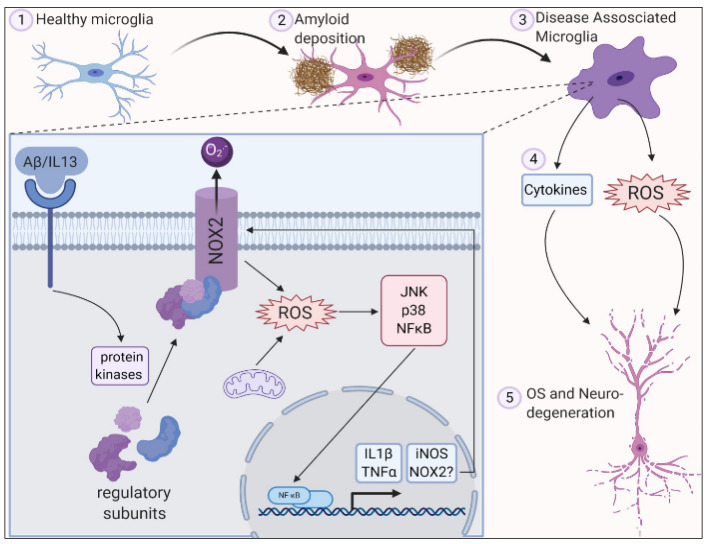
ROS is generated in microglia by activation of NADPH oxidase 2 along with deposition of amyloid plaques and inflammation leading to oxidative stress and neurodegeneration. Reproduced with permission from Simpson et al. [10].

In Alzheimer’s disease (AD), oxidative stress biomarkers such as protein carbonyls and 3-nitrotyrosine, 8-hydroxydeoxyguanosine, 8-hydroxyguanosine, 4-hydroxynonenal, and malondialdehyde were shown to increase in the blood. An alteration in the expression of antioxidant enzymes such as superoxide dismutase and catalase was reported to be the cause of familial AD [67]. In AD, β-amyloid is produced in increased quantities causing mutations primarily in the amyloid precursor protein (APP), and presenilin-1 (PS-1), and presenilin-2 genes. β -amyloid level increases with age in human beings [68]. This age-dependent accumulation of β-amyloid was studied by Anantharaman et al. [68] using APP/PS1 transgenic mice. Brain sections were analyzed in the 3-, 6-, 9-, 12-, and 14-month-old mice. There was no deposition of β-amyloid in the 3-month-old mice while 12-month-old mice brains had greater deposition in the neocortex and hippocampus. By 14 months, β-amyloid deposits were seen in small blood vessel walls in the brain and leptomeninges suggesting age-related deposition of β-amyloid in APP/PS-1 mice. It was also observed that the activity of the superoxide dismutase enzyme was significantly decreased when compared to wild-type mice resulting in mitochondrial dysfunction, oxidative stress, and apoptosis. It could therefore be concluded that aging causes an increase in β-amyloid levels leading to the inactivation of superoxide dismutase enzyme and thereby increasing ROS levels which can lead to neuronal death associated with oxidative stress (Figure 1).

In Amyotrophic Lateral Sclerosis (ALS), both upper and lower motor neuron loss is seen which results in muscle wasting, weakness, and paralysis. Its etiology is unknown but superoxide dismutase enzyme mutations are observed [69]. Mutation in the superoxide dismutase-1 enzyme forms protein aggregates which cause loss of enzymatic function. Superoxide dismutase-1 enzyme plays an important role in the clearance of free oxygen radicals. With the loss of enzymatic function in ALS, the accumulation of oxygen radicals increases leading to oxidative stress.

## 4. Antioxidants in CNS and PNS

**Figure 2:**
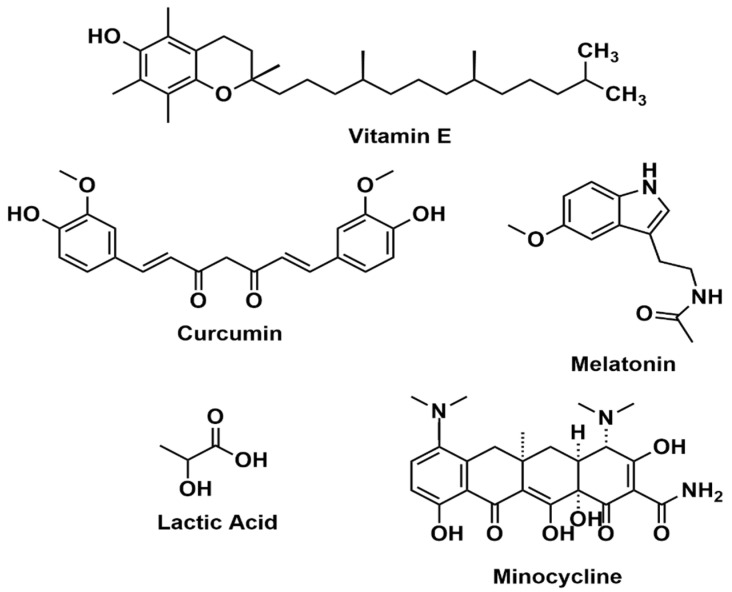
Chemical structures of exogenous antioxidants used in PNS and CNS.

Antioxidants help in reducing free radical accumulation and thereby reducing oxidative stress. These antioxidants can be administered either as a drug or by incorporating them with other biomaterials to form hydrogels or nanofibers. Direct administration of the drug can be performed orally, intraperitoneally or via the intragastric route. Various compounds used as direct administration have been described below. Figure 2 shows structures of some of the antioxidants used in CNS and PNS, discussed in this section.

### 4.1. Vitamin E

Vitamin E is a family of eight naturally occurring members of fat-soluble compounds composed of tocopherols and tocotrienols [70]. Out of the eight members, α-tocopherol is the most biologically active compound as it has a greater affinity of tocopherol transfer protein. It consists of an electrophilic hydroxyl group on the chroman ring to scavenge the oxygen-free radicals. It is reported to be a lipid-soluble antioxidant that helps to maintain the integrity of the cell membrane which can change due to PUFA generated oxygen-free radicals [71].

α-tocopherol is known to be an essential nutrient for humans, it is not naturally present in the body and has to be supplemented through nutrient sources such as peanuts [29], almonds [72], and sunflower oil [73]. α-tocopherol is transported from the liver by ATP-binding cassette proteins such as ATP-binding cassette transporter A1 and ATP-binding cassette transporter G into two types of lipoproteins namely very low-density lipoprotein and low-density lipoprotein particles which circulate in the extracellular fluids. It is then enzymatically exchanged by transfer proteins to high-density lipoprotein (HDL). This α-tocopherol bound HDL is transported by tocopherol transport protein to the CNS by penetrating through the blood–brain barrier (BBB). It is reported that in neurodegenerative diseases such as AD, PD, and Down’s syndrome, where ROS concentration is intrinsically high, tocopherol transport protein concentration is also increased which in turn increases the transport of α-tocopherol in the CNS [74]. Therefore, α-tocopherol can be used to ameliorate ROS production in neurodegenerative disease through vitamin E supplementation. Liu et al. [75] studied the effect of vitamin E on brain damage caused due to environmental pollutants mainly particulate matter (PM), gases, organic compounds, and metals. Amongst these, the most commonly encountered pollutant in indoor and outdoor air is PM 2.5 and formaldehyde (FA) which resulted in cognitive deficits, pathological brain alterations, β-Amyloid accumulation, glia activation, and oxidative stress. The authors studied the effects of co-exposure of FA and PM 2.5 in SPF male C57BL/6 mice. The mice were exposed to the mixture of FA and PM 2.5 by intranasal instillation and vitamin E was given by intragastric administration of dose 50 mg/kg/day for 7 days and studied for brain damage. It was observed in histological studies that after co-exposure, the pyramidal cells in the cornu ammonis region of the hippocampus became loose and disordered with swollen and deformed shape and with vitamin E administration, most pyramidal cells remained intact. The administration of vitamin E increased the cell number in cerebral and prefrontal cortex as compared to co-exposure control group. The expression of proteins such as Aβ, Tau-P, and GFAP was reduced with vitamin E administration in comparison with co-exposure group. It was also observed that ROS and COX-2 levels decreased and gutathione levels and superoxide dismutase activity was higher in vitamin E administered group than the co-exposure group indicating the antioxidant property of vitamin E. The antioxidant efficacy of vitamin E was also shown in high fat diet (HFD) associated with oxidative stress. The consumption of HFD releases excessive amount of free radicals [76,77,78], which was shown to cause cardiovascular and metabolic diseases and increase the risk of neurological deficits in different regions of the brain [79]. The role of vitamin E supplemented diet on reducing epileptic seizures in male Wistar rats was studied by Alzoubi et al. [80]. In this study, rats were fed HFD in combination with 100 mg/kg of vitamin E supplement once daily for 6 weeks using oral gavage. The results concluded that vitamin E prevented oxidative damage by strengthening the antioxidant mechanism in the hippocampus and cortex of rats subjected to an HFD thereby reducing the seizure threshold. This reduction was due to the protective mechanism of vitamin E against reduced levels of glutathione/oxidized glutathione ratio and catalase at the hippocampus. Additionally, the scavenging activity of vitamin E against reactive nitrogen species and lipoperoxyl radicals, inhibiting cyclooxygenase- and 5-lipooxygenase-catalyzed eicosanoids lead to reduced oxidative stress.

### 4.2. Curcumin

Curcumin is a natural polyphenol component obtained from *Curcuma longa*. It consists of two aromatic rings containing phenolic groups linked by seven carbon spacers consisting of diketone moiety [81]. The phenolic group present in curcumin is reported to have the ability to react with free radicals to reduce oxidative stress. It is also known to increase the expression of antioxidant enzymes such as superoxide dismutase, catalase, a glutathione reductase. Samarghandian et al. [82] studied the effect of restraint stress on the brain. When the body experiences physiological stress, cells require high energy to adapt to the abnormal environment which increases metabolic rate causing excessive generation of free radicals leading to oxidative stress. The authors used a rodent restrainer made of plexiglass which closely fits the rat’s body to induce restraint stress in Wistar albino rats to evaluate the antioxidant property of curcumin. Curcumin was administered intraperitoneally at doses of 10 mg/kg, 20 mg/kg, and 30 mg/kg for 21 days. It was observed that following restraint stress, the level of glutathione, superoxide dismutase, glutathione peroxidase, glutathione reductase, and catalase activities were reduced. During the first two weeks post-treatment with 30 mg/kg curcumin, the oxidative stress parameters such as levels of glutathione, superoxide dismutase, glutathione peroxidase, glutathione reductase, and catalase reduced and after 3 weeks post treatment at the same dose the parameters increased indicating the antioxidant capacity of curcumin.

Rocha-Ferreira et al. [83] studied the pathophysiology of neonatal hypoxic–ischemic encephalopathy (HIE). HIE brain injury is estimated to cause around 1 million deaths worldwide, and can also result in lifelong disabilities such as cerebral palsy and seizures [84,85]. The HIE brain damage causes initial energy loss causing the reduced supply of oxygen and glucose to the cells leading to reduced mitochondrial oxidative phosphorylation, reduced adenosine triphosphate level triggering excitotoxicity, neurotoxicity, and oxidative stress [84,86,87,88]. The oxidative stress induced by HIE brain injury is known to cause an increase in an enzyme called inducible nitric oxide synthase [89,90,91]. Inducible nitric oxide synthase (iNOS) is one of the isoforms of nitric oxide synthases, which is responsible for the synthesis of nitric oxide from the amino acid, L-arginine [89,92,93]. It is expressed when the cells are exposed to inflammatory cytokines. In HIE brain injury, inducible nitric oxide synthase expression increases in the brain causing increased production of nitric oxide [94,95]. Nitric oxide which is a lipophilic diatomic molecule interacts with oxygen free radicals resulting in the formation and accumulation of peroxynitrite causing oxidative damage [90,93]. In the study by Rocha-Ferreira et al. [83], moderate to severe brain damage was induced in P7 mice by exposing them to a hypoxic chamber. The injured mice were treated with curcumin via intraperitoneal injection at doses of 20, 44, 100, 200, and 400 µg/µL in DMSO after a 60 min delay following a 60 min hypoxic–ischemic insult. Histological studies revealed that 48 h post curcumin treatment caused a significant reduction in ipsilateral forebrain tissue loss, brain volume loss in pyriform cortex and striatum, ipsilateral reactive astrogliosis, and microglial activation. A decrease in expression of inducible nitric oxide synthase in the hippocampus after administration of curcumin was reported by the authors indicating a reduction in oxidative stress.

The effect of curcumin and propolis was comparatively analyzed by Yüce et al. [96] on adult female Wistar albino rats with sciatic nerve injury. The rats were treated with 100 mg/kg curcumin and 200 mg/kg propolis through a nasogastric tube for 28 days. The study concluded that curcumin and propolis showed better results in walking track and electrophysiological analyses compared to methylprednisolone control. This was because curcumin has antioxidant, anti-inflammatory, neuroprotective, and angiogenic properties. The positive results shown after the administration of propolis were attributed to the presence of caffeic acid phenethyl ester (CAPE) as an active component. CAPE provides protection against lipid peroxide, decreases malondialdehyde and nitric oxide levels, and increases glutathione and superoxide dismutase levels and thereby reducing the ischemic effect on the brain and increasing neuroprotective properties.

### 4.3. Melatonin

Melatonin, chemically known as *N*-acetyl-5-methoxytryptamine, is an effective antioxidant that prevents peroxidative injuries. It also reduces the harmful effects of free radicals by reducing nitric oxide synthase activity. It stimulates superoxide dismutase, glutathione peroxidase, and glutathione reductase, which are enzymes that help in the breakdown of free radicals [97]. In a study performed by Gul et al. [98], low and high doses of melatonin were administered after a blunt sciatic nerve trauma in young male Wistar albino rats. The treatment groups received 30 mg/kg, 50 mg/kg and 100 mg/kg melatonin. When melatonin was administered intraperitoneally immediately after 24 h clip compression spinal cord neurotrauma, minimal intracellular edema in white matter, neutrophil infiltration, and lamellar detachment of axonal myelin was observed. This was due to the very strong lipophilic and hydrophilic property of melatonin which promotes its penetration into cellular and nuclear membrane protecting the nuclear components and cellular organelles against free radicals.

Baydas et al. [99] studied neurotoxic effects of industrial thinners (with toluene as the major component) in adult male Wistar rats. Chronic abuse of toluene causes structural and functional damage to the organs [100,101,102]. It can cause encephalopathy and may lead to irreversible damage in the brain structure leading to neural dysfunction. Toluene inhalation enhances astrocyte activation and when there is a trauma to the brain, it leads to neuronal damage [103,104,105]. An increase in the number of activated astrocytes was characterized by overexpression of the glial fibrillary acidic protein. This increase in the number of astrocytes is known as reactive gliosis which leads to the formation of the glial scar. Baydas et al. [99] administered melatonin to adult male Wistar rats that were exposed to thinner in whole-body inhalation chambers. The thinner exposed rats were divided into two groups. One of the thinner exposed groups was given melatonin at a dose of 10 mg/kg daily intraperitoneally for 45 days. It was concluded that after melatonin treatment, the levels of lipid peroxidation products such as malondialdehyde and 4-hydroxyalkenals were reduced and glutathione levels were elevated in comparison with the control and thinner exposed group without melatonin treatment suggesting the antioxidant property of melatonin.

### 4.4. Nanoceria

Cerium oxide nanoparticles or nanoceria have found application as a pharmacological agent, in drug delivery, and scaffolding due to their unique properties such as antioxidant activity and ability to self-regenerate their surface for ROS scavenging [106]. These unique properties are due to the blue shift exhibited by nanoceria in the ultraviolet absorption spectrum, the shifting and broadening of Raman allowed modes and lattice expansion. Its antioxidant property is due to the presence of two oxidation states Ce^+3^ and Ce^+4^ which increase oxygen vacancies thereby acting as a catalyst for oxidation and reduction reactions. Das et al. [107] synthesized 2–5 nm diameter nanoceria using a microemulsion process and investigated the neuroprotective efficacy in primary neuronal and glial cells extracted from rat spinal cords. The neurons in the nanoceria treated culture exhibited normal electrical activity as compared to the control culture demonstrating retention of normal function. Furthermore, nanoceria pre-treated neurons exhibited significantly higher cell survival when exposed to H_2_O_2_ induced oxidative injury compared to untreated cells. It was observed that the peroxide detoxification ability of the cells was enhanced upon treatment with nanoceria, providing protection against ischemic insult, thus increasing neuronal survival. The pre-treated glial cell population did not show any difference in comparison with the control group.

### 4.5. Lactic Acid

Lactic acid is a weak acid formed as a byproduct of anaerobic respiration. It is dissociated under physiological conditions into hydrogen ions and lactate ions [108]. Both lactic acid and lactate ions enter cells and penetrate through the lipid membrane. Lactic acid functions as an antioxidant by scavenging reactive oxygen species such as superoxide and hydroxyl radicals, and inhibiting lipid peroxidation [109]. Lampe et al. [110] studied the antioxidant property of lactic acid since it degrades when exposed to water into natural metabolites. Oxidization of lactic acid produces byproducts such as pyruvate, which also has the antioxidant property of scavenging hydrogen peroxide and superoxide radicals [111]. The scavenging activity of lactic acid was demonstrated by preparing a solution containing lactic acid, photoinitiator, and luminol. This solution was then added to the cell cultures of primary neural cells obtained from embryonic day 14–15 (E14–E15) Sprague-Dawley rat embryo-fetal forebrain cells. The photoinitiator, irgacure 2959 (1-[4-(2-hydroxyethoxy)-phenyl]-2-hydroxy-2-methyl-1-propane-1-one), was exposed to UV light of 365 nm wavelength to generate free radicals. The effect of lactic acid on free radicals was examined by adding luminol to the solution which gets oxidized in the presence of free radicals and produces 3-aminophthalate which is a light emitting species. The number of free radicals is proportional to the light emitting species which were quantified using luminometer. The addition of lactic acid in primary neural cell cultures resulted in reduced number of free radicals when compared to cells cultured in the absence of lactic acid. The authors also observed that when lactic acid was added to neural cell cultures in the absence of free radicals, there was no influence on ATP production in cells. The addition of lactic acid in the presence of free radicals increased the ATP content of the cells thereby improving the cell viability. This was likely due to free radical scavenging effect of the lactic acid. The exposure of cells to lactic acid for 5 days increased the total DNA content and nestin gene expression indicating its antioxidant property.

### 4.6. Hydroalcoholic Extract of the Red Propolis (HERP)

Propolis is obtained from honeybees and red propolis is its Brazilian variant with various properties such as anti-inflammatory, antioxidant, antimicrobial, and anti-carcinogenic [96,112,113]. The constituents of red propolis include isoflavones such as formononetin, pinocembrin, vestitol, and biochanin A [112,113,114,115,116]. Barbosa et al. [117] extracted HERP using propolis and ethanol at room temperature and studied its composition using high-resolution mass spectrometry. HERP was administered in male Wistar rats with a left sciatic nerve injury to analyze its antioxidant properties. In the sham group, rats were anesthetized and the left sciatic nerve was exposed without nerve crush injury. HERP was administered orally to the treatment group at the doses of 1 mg/kg and 10 mg/kg once a day for 28 consecutive days. The two control groups were sham-operated and administered either with vehicle (2% *v*/*v* Tween 80 in saline) or the highest dosage of HERP (10 mg/kg). Behavioral assessment was carried out at different time points and results demonstrated that Basso, Beattie, and Bresnahan test, sciatic function test, and beam walk performance were enhanced in the 10 mg/kg dose treatment group indicating the neuroprotective effect of HERP. The histological examination of the sciatic nerve concluded that the mean axon number increased, and cellular inflammatory infiltrate decreased in the treatment group administered with 10 mg/kg HERP when compared to the vehicle-treated control group. It was concluded that compounds present in HERP, such as formononetin and biochanin A, were responsible for the neuroprotective and antioxidant properties. The observed neuroprotective and antioxidant properties can be explained by the ability of (a) formononetin to increase transcription of pro-neural genes via neurogenin-2 promoter activity [118], and (b) biochanin A to inhibit microglial activation and proinflammatory factor generation in reducing oxidative stress [119].

### 4.7. Minocycline

Minocycline is a clinically used antibiotic and anti-inflammatory drug [120]. It is a second-generation, semi-synthetic tetracycline, highly lipophilic drug which can cross the BBB [121] leading to its accumulation in cells of the cerebrospinal fluid and the CNS [120,122]. Additionally, it is an antioxidant with an anti-apoptotic property. Minocycline is clinically available as minocycline hydrochloride (MH), which is a water-soluble small molecule drug that degrades in an aqueous solution at room temperature and 37 °C in the presence of fetal bovine serum [123]. The water-soluble nature of MH and its low efficacy when delivered as nanoparticles incorporated in polymers such as poly(lactic-*co*-glycolic acid) [124,125] and polycaprolactone [126,127] resulted in drug release which lasted for only 24 h. To obtain a slow and sustained release of MH over a period of time and to enhance the antioxidant property of MH, Wang et al. [128] synthesized dextran sulfate (DS)-MH complex. DS is a negatively charged biocompatible polysaccharide with a high affinity to metal ions such as Ca^2+^ and Mg^2+^. These ions can induce the formation of an insoluble complex of DS-MH which was synthesized in this study by supplementing the solvent with MgCl_2_. This complex was then encapsulated in injectable agarose hydrogel to maintain sustained and localized release of the drug in the intrathecal space at the injury site. In vitro release of MH was assessed every 24 h by injecting the agarose hydrogel on a culture dish and observing its release under UV absorbance at 245 nm. MH released on day 44 was added to rat cortical neurons isolated from E17 rat embryos. At a dose of 1 mg MH/mL hydrogel, it was observed that the drug release was slow over 37 days. When the MH loading was increased to 3 mg/mL, the drug release rate was accelerated during the acute stage. In vivo study was conducted to evaluate neuroprotective effects of DS-MH hydrogel in female Sprague Dawley rats with unilateral cervical contusion injury at the C5 level. It was observed that a dose of 1.3 mg/kg over 21 days resulted in reduced chronic inflammation, delayed apoptosis, and enhanced neuroprotection. Neuron survival was increased 3-fold and more myelinated axons were preserved when compared with the control group. Minocycline targeted the resident microglia in the injured spinal cord leading to selective inhibition of classical activation (M1) microglia polarization.

### 4.8. Nitrones

Nitrones are synthetic antioxidants, which trap and scavenge free radicals by forming covalent interactions. Considerable efforts have been made to prepare analogs of spin-trapping agent alpha-phenyl-tert-butyl nitrone (PBN), which was shown to be highly effective in reducing oxidative induced damage in in vivo stroke (ischemia/reperfusion insult) models. However, after the failure of PBN analog, NXY-059 from AstraZeneca in advanced stroke clinical trials, research in the domain became dormant. The weak radical trapping activity, low BBB penetration and inadequate trial design were cited to be the reasons behind the failure of NXY-059 in phase III clinical trials. Recently, there has been a renewed interest in developing PBN analogs as radical scavengers in neuroprotective applications (including neurodegenerative diseases) [44,129,130,131]. However, despite some promising results in in vitro and in vivo rodent models, considerable research is needed to establish their effectiveness for clinical translation, in particular, their bioavailability in the brain. This topic has been central to multiple recent reviews and therefore, we would like to draw the attention of readers to these reviews [130,132,133,134].

## 5. Bioengineering Approaches to Regenerating CNS and PNS

Regeneration and repair of CNS and PNS present many challenges as both the systems have limited regeneration ability following trauma. PNS injury causes impairment in the axoplasmic flow due to Wallerian degeneration that occurs at the distal end of the injury [135,136]. The proximal part of the axon regenerates forming synaptic connections with the adjacent tissues. This immune response is mediated by Schwann cells and macrophages which play an important role in clearing the debris caused by injury [135,137]. Schwann cells also secrete neurotrophic factors, cytokines, cell adhesion molecules, and extracellular matrix components for the regeneration process to occur [137,138,139]. Repair after injury constitutes preservation of surviving cells, regeneration of axons, and rehabilitation of neural functions. Cell therapy and bioengineered scaffolds were used to improve the regeneration and repair of neurons [140,141,142,143,144]. The field of neural tissue engineering focuses on improving the environment that forms a bridge between the cells and the biomimetic scaffold in order to repair and reinstate neural tissue function. Three-dimensional scaffolds and other scaffold-like materials were used to provide physical support for nerve regeneration [145,146,147,148,149,150,151]. Given the active role of ROS in neurotrauma and in neurodegenerative diseases, different bioengineering strategies have been developed to deliver antioxidants to neutralise ROS both in vitro and in vivo models. Common bioengineering approaches include two- and three-dimensional bio scaffolds to promote cell growth environment and simultaneously deliver antioxidants to mitigate ROS. In this section, different bioengineering approaches developed to date are discussed.

### 5.1. Antioxidants Derived from Natural Sources

Chitosan (CS) is a natural biomaterial that has been extensively used for nerve repair. It is obtained from the deacetylation of chitin which is a natural polysaccharide derived from exoskeletons of crustaceans [152,153]. It is biocompatible, supports axon regeneration, reduces scar formation, and has anti-inflammatory and antioxidant properties. Its degradation products are also known to positively affect the regeneration process of nerves [154]. Boido et al. [155] used chitosan-based hydrogels as cell carriers for mesenchymal stem cells (MSCs) for the treatment of SCI. The chitosan-based hydrogel was synthesized by adding β-glycerophosphate disodium salt hydrate (β-GP) to the chitosan-HCl solution. The addition of β-GP reduced the gelation time up to 5 min and increased the pH values of the hydrogel up to 7.11 ± 0.03. In vitro analysis was carried out using MSCs obtained from BCF1 mice which express an enhanced green fluorescent protein (EGFP) under β-actin promoter. SH-SY5Y neuroblastoma cell line was plated inside or near a 100 µL drop of the hydrogel while enhanced green fluorescent protein transfected MSCs were grown inside a 100 µL drop of chitosan/β-GP hydrogel to assess the cell viability. It was observed that SH-SY5Y cells were viable at 7 days in vitro and also underwent mitosis indicating that the hydrogel was cytocompatible with the cells. The enhanced green fluorescent protein transfected MSCs showed fibroblast-like morphology within the hydrogel indicating good hydrogel biocompatibility. SH-SY5Y cells were also cultured under oxidative stress and dichlorodihydrofluorescein diacetate assay was used to measure the level of stress. It was observed that chitosan/β-GP hydrogel along with MSCs showed a reduction in cellular oxidative stress. In vivo study was conducted in C57BL/6J mice with complete spinal cord transection injury. The injury site was injected with enhanced green fluorescent protein transfected MSCs embedded in the chitosan/β-GP hydrogel. After 1 week, the lesion site showed the presence of MSCs both within and around the lesion reducing the lesion volume and improving functional recovery. Chitosan was also loaded with cerium oxide nanoparticles to be used in the treatment of SCI by Fang et al. [156]. Inorganic nanoparticles such as cerium oxide when loaded in natural biopolymers are known to have autocatalytic, biocompatibility, antioxidant, and anti-inflammatory properties [106,157,158]. Cerium oxide nanoparticles protect against cellular damage caused by free radicals due to the presence of shielded 4f electrons [159]. When used in combination with CS, the toxicity of the nanoparticles is reduced and their solubility is increased. Fang et al. [156] isolated spinal cord cells from adult rats and cultured in vitro, in the presence of CS-filled cerium oxide nanoparticles. Increased cell viability and decreased cell death were observed due to the presence of Ce^3+^ and Ce^4+^ valence states resulting in free radical scavenging property of cerium nanoparticles thereby reducing oxidative stress. Azizi et al. [160] also reported the use of CS, vitamin E, and pyrroloquinoline quinone (PQQ) to treat peripheral nerve injury in male white Wistar rats. The left sciatic nerve was transected in these rats leaving a gap of 10 mm. The control group was implanted with a CS conduit. The treatment groups consisted of rats implanted with CS conduit filled with 20 µL vitamin E, 20 µL PQQ, and a combination of 10 µL of vitamin E + 10 µL PQQ. The analysis was performed at 4-, 8-, and 12-weeks post-implantation. It was observed that sciatic nerve function recovery in the treatment group had a better mean value than the control group. The functional recovery was higher in conduits with both vitamin E and PQQ. The treatment group with both vitamin E and PQQ also showed increased conduction velocity of regenerated nerve, gastrocnemius muscle mass, nerve fibers, axon diameter, and myelin sheath thickness in comparison with the control group as well as the treatment group consisting of only vitamin E and PQQ. The authors observed that SC have the ability to adhere and proliferate on CS, thereby stimulating tissue repair and angiogenesis. It was also observed that vitamin E which is a lipophilic antioxidant, protected cells against lipid peroxidation and improved nerve function which was affected due to oxidative stress. PQQ oxidized the redox modulatory site of N methyl D-aspartate receptors which induces cell injury by polysynaptic pathways. PQQ also increased nerve growth factor synthesis to accelerate nerve regeneration. It was concluded that the combination of vitamin E and PQQ resulted in synergistic effect on nerve regeneration. Singh et al. [37] reported the use of a polyurethane (PUAO) conduit filled with CS-gelatin (CG) cryogel filler. PUAO is a biodegradable elastomer formed by the combination of polycaprolactone and poly (ethylene glycol). It is a flexible, biocompatible material with antioxidant properties [161,162]. It was electrospun to fabricate a hollow nerve guidance conduit (NGC). CG cryogel was filled unidirectionally into the hollow conduit. CG cryogel was synthesized using modified cryogelation technology which allowed the solvent to freeze in one direction leading to the formation of pores in a single direction with optimum size for axonal regeneration. Cryogel was cross linked using glutaraldehyde. In vitro biocompatibility test for electrospun PUAO fibers and NGC was carried out using neuro 2a cells (mouse neural crest-derived cell line) which showed enhanced proliferation of these cells on the conduit over a period of 7 days with no cytotoxicity. Further to evaluate the efficiency of the conduit, rat bone marrow stem cells were isolated in vitro and cultured over the conduit. Cell adhesion, and proliferation rate was maximum at the end of 10 days. It was noted that the conduit could serve as suitable matrix for stem cell transplantation for in vivo nerve regeneration and the authors concluded that due to presence of PUAO and CS in a single conduit, cell proliferation was enhanced and showed potential to be used in vivo peripheral nerve regeneration. Moattari et al. [163] reported the use of curcumin and chitosan membrane for peripheral nerve injuries. Curcumin is obtained from a plant named *Curcuma* which is known to have anti-inflammatory and antioxidant properties. Curcumin was also reported to improve motor functional recovery and promote nerve regeneration. Chitosan membrane was prepared as thin films and 1 × 1 cm patches were cut. These patches were implanted in male adult Wistar rats with the transected sciatic nerve. The treatment groups consisted of intraperitoneal administration of 100 mg/kg/day curcumin for 4 weeks, implanted chitosan patches, and combination of chitosan patch with intraperitoneal administration of 100 mg/kg/day curcumin for 4 weeks. It was observed that sciatic functional index and withdrawal reflex latency were improved in the animals with the combination treatment over a period of 8 weeks post-surgery. Histological examination of the sciatic nerve also concluded that the increase in number of myelinated nerve fibers, size of nerve fiber and myelin thickness when compared to treatment with only curcumin or chitosan patch. It was concluded that oxidative stress followed by sciatic nerve injury was reduced by administration of curcumin leading to inhibition of apoptosis and reduced neuron loss. Chitosan enhances production of growth factor b1, platelet-derived growth factor, and interleukin 1 simulating macrophages. These macrophages down-regulate pro-inflammatory cytokines and up-regulate anti-inflammatory cytokines thereby preventing scar formation and provided suitable microenvironment for axon regeneration.

Another natural biomaterial, lignin was also explored by researchers for its antioxidant property [164,165,166,167,168]. It is a type of organic polymer present in the tissues of most plants. They play an important role in the formation of cell walls, especially in wood and bark [169,170,171]. It fills the spaces in the cell wall between cellulose and pectin. In a study performed by Amini et al. [172], lignin nanoparticles were incorporated in PCL electrospun nanofibers for application in nerve tissue engineering. PCL-lignin nanofibers were fabricated with 0, 5, 10, and 15% lignin concentration, and PC12 cells and human adipose stem cells were cultured in vitro over the scaffold. It was observed that with the increase in lignin concentration, the cell viability of both the cell lines increased in comparison to the control samples. It was also observed that 10% lignin concentration promoted cell viability and 15% lignin concentration decreased cell viability. The cells also showed gradually elongated neurite outgrowth with the increase in lignin concentration. The PCL-lignin hollow conduit was prepared by rolling it around the steel wire of desired dimensions. This conduit was implanted in adult male Wistar rats with a left sciatic nerve defect of 10 mm in length. At 6 weeks post-implantation, there was no difference in sciatic functional index and motor functional index in both autograft and the nerve conduit with 15% lignin group. Increased collagen fiber area, reduced muscle fiber diameter, and lower wet gastrocnemius muscle weight was observed in conduit groups with 10 and 15% lignin concentration indicating a positive effect of lignin. The presence of S100, βIII-tubulin and Map-2 markers in the immunohistochemical staining of the conduit indicates the positive effect of lignin on Schwann cells. The authors concluded that the positive effect of lignin was due to the presence of methoxyl and hydroxyl functional groups which inhibit oxidative reaction by donating hydrogen. PCL/lignin nanofibers were also studied by Wang et al. [173] Schwann cells were seeded on the electrospun nanofibers and was exposed to H_2_O_2_ oxidative insult for 20 min which increases intracellular levels of ROS leading to oxidative stress. Increased cell viability and proliferation, increased cell density and spindle shape with bipolar extension were observed in PCL/lignin nanofiber group than pure PCL group on day 1 and day 3 suggesting that lignin exerted a protective effect against H_2_O_2_ toxicity by providing hydroxyl and methoxyl functional groups to hydrogen to terminate oxidation propagation reaction and inhibit cell death caused by oxidative stress. DRG neurons were also isolated from the embryo of 14-day-pregnant rats and seeded on the nanofibers to observe the neurite growth. The average neurite length on PCL group was 46.75 ± 15.63 μm and 108.83 ± 21.45 μm on PCL/lignin nanofiber group indicating that lignin improved biocompatibility and antioxidant properties of the nanofibers which promoted neuronal adhesion, proliferation and growth.

Samadian et al. [174] used two natural biomaterials namely collagen and naringin to prepare hydrogel to be used as a scaffold for sciatic nerve regeneration. In vitro studies were performed by placing hydrogel in a 96-well plate and seeded with SCs resulting in the proliferation of cells and increased cytocompatibility. The hydrogel was injected into the left sciatic nerve injury site of adult male Wistar rats which resulted in high healing efficacy, improved thermal pain sensitivity, and reduced gastrocnemius muscle wet weight loss. On histological examination of muscle fibers, it was observed that collagen fibers and some degree of disorganization were present in groups injected with collagen only, while the groups injected with collagen/naringin hydrogel exhibited the lowest fibrosis, less muscular shrinkage and better healing of gastrocnemius muscle. It was observed that the incorporation of naringin efficiently improved the healing process of the injured nerve. Naringin is a glycoside extract obtained from fruits such as grapefruit, tomato, and citrus fruits which has anti-inflammatory, anti-apoptotic, anti-osteoporotic, anti-carcinogenic, and neuroprotective properties. It was concluded by the authors that the neuroprotective effect of naringin is due to its ability to inhibit neural apoptosis and enhance the expression of vascular endothelial growth factors and brain-derived neurotrophic factors.

Melanin, a biologically derived pigment known for its antioxidant property, electrical property and photoconductivity [175,176,177,178,179,180,181,182] was incorporated in silk fibroin to fabricate electrospun nanofibers by Nune et al. [183]. The nanofibers with both aligned and random fibers were synthesized and cut into small dimensions to be placed in a 96-well plate. In vitro cell culture was performed using SH-SY5Y cells and it was observed that cell viability, adhesion, and proliferation increased gradually over 7 days on both aligned and random nanofibers. It was also observed that radical scavenging activity was greater in silk/melanin nanofibrous scaffolds when compared with plain silk fibroin scaffolds which confirmed the positive role of melanin in reducing oxidative stress.

Melatonin, a potent antioxidant as mentioned earlier, promotes the expression of antioxidant enzymes such as superoxide dismutase, glutathione peroxidase, glutathione reductase, and catalase [39,184,185], was studied by Qian et al. [186]. Nerve guidance conduit using melatonin (MLT) and PCL was synthesized by spraying PCL and MLT onto a tubular mold. Cell culture studies were carried out on the conduit using rat Schwann cells. The cells were subjected to oxidative insult using H_2_O_2_. It was observed that cell proliferation increased, and cells exhibited protuberances on MLT/PCL conduit than PCL conduit. It was also noted that there was a significant increase in expression of antioxidant stress markers such as manganese superoxide dismutase, glutamate cysteine ligase catalytic, and heme oxygenase-1 on MLT/PCL conduit than on PCL. In vivo studies were carried out in male Sprague Dawley rats with a right sciatic nerve defect of 15mm long. The conduit was sutured to both the ends of the nerve. It was observed that sciatic functional index, nerve conducting velocity, regenerated axon area, myelinated axons number, average myelinated axon diameter, and myelin sheath thickness were increased in MLT/PCL conduit when compared with autografts at 6 and 12 weeks post-operatively. Manganese superoxide dismutase and glutamate cysteine ligase expression was increased in MLT/PCL conduit than autograft and PCL groups at 12 weeks post-operatively suggesting that the reduction in oxidative stress was due to the presence of melatonin.

Salles et al. [187] synthesized a biodegradable compound consisting of an antioxidant compound and mineral salts. The antioxidant compound consisted of β-carotene, α-tocopherol, vitamin B complex, selenium salts, zinc salts, magnesium salts, phosphor salts, glutamic acid, soy lecithin, hydrolyzed collagen, glycosaminoglycan sulfate, and chondroitin sulfate. The final consistency of the compound was similar to epoxy resin making it easier for local application. In vivo study was performed in Wistar rats with right sciatic nerve injury. The injured segments were approximated and sutured and the antioxidant compound was placed at the injury site. It was observed macroscopically that 30 days after application of the compound, the integrity of nerve strand and nerve neoformation under the suture wire was present. It was also observed that there was the absence of nerve degeneration, connective tissue formation, and fibrotic tissue under the suture wire when compared to the control group consisting of suture approximation without application of the antioxidant compound. It was concluded that the antioxidant compound reduced the oxidative stress that occurred during the injury and promoted nerve regeneration.

### 5.2. Synthetically Obtained Antioxidants

A new generation of synthetic biomaterials is being developed and widely used as their composition, properties and shape can be controlled and therefore can be easily reproducible. Nanoceria as mentioned earlier in the review has antioxidant properties which were combined with gelatin to prepare nanocomposite to synthesize a nerve conduit by Marino et al. [188]. Gelatin-nanoceria nanofibrous scaffolds were fabricated using the electrospinning method to obtain randomly oriented and aligned fibers. The scaffolds were cut into pieces on which SH-SY5Y cells were cultured in vitro. ROS produced by the cells was measured by using 20, 70-dichlorofluorescein diacetate in serum-free Dulbecco’s modified eagle medium for 30 min at 37 °C in the presence and absence of the scaffolds. It was observed that ROS levels in cells seeded on the gelatin/nanoceria fibers were less than cells seeded on plain gelatin fibers indicating the scavenging activity of nanoceria. Histological examination of cells seeded on the aligned gelatin/nanoceria fibers showed the presence of elongated nuclear shape in the direction of the fibers and also increased cell growth, adhesion, and proliferation. Nanoceria was also studied by Qian et al. [189] by fabricating PCL/collagen/nanoceria (PCL/COL/NC) nerve conduit by layer by layer 3D manufacturing. A rolling tube mold was used on which three layers were sprayed. The inner layer consists of NC/PCL, the middle layer consisted of only PCL and the outermost layer consisted of only collagen. In vitro cell culture on the nerve conduit was performed using rat Schwann cells. It was observed that expression of S100, β-III-tubulin (Tuj1), myelin basic protein, and glial fibrillary acidic protein (GFAP) markers were higher, cells exhibited protuberances, improved cellular adhesion, and elongation in PCL/COL/NC treated cells than in PCL conduit. The rat Schwann cells cultured on PCL/COL/NC conduit was subjected to H_2_O_2_ oxidant insult and it was observed that the antioxidant markers such as manganese-dependent superoxide dismutase, glutamate-cysteine ligase catalytic were increased when compared to PCL conduit. This was reported to be due to the presence of nanoceria which scavenges intracellular ROS and reduces DNA damage. PCL/COL/NC nerve conduit was implanted into male Sprague Dawley rats with 15 mm long sciatic nerve defect and their performance was evaluated at 6-, 12-, and 18-weeks post-surgery. It was observed that sciatic function index was high, improved wound healing, high gastrocnemius muscle weight, and absence of ulcers at 12- and 18-weeks post-surgery when compared to the autografts. Nerve conduit showed higher expression of nuclear factor-like 2 (Nrf2), heme-oxygenase-1 (HMOX1), tumor necrosis factor α (TNFα), and interleukin 6 (IL6) when compared to autografts and PCL conduit. It was concluded that PCL/COL/NC conduit significantly reduces oxidative stress and inflammation after peripheral nerve trauma.

Another synthetic biomaterial that was designed by Wang et al. [190] was a low-cost gel consisting of tannic acid and pluronic F-127 (TA/PF gel). The prepared gel was placed in vivo in two different animal models. The first model was the rabbit dura injury model in which craniotomy was performed to expose the brain dura and was sutured, fascia implanted, and sealed with the TA/PF gel. In the second model, a mouse with an exposed thoracic spinal cord was used which was sutured and sealed with TA/PF gel. It was observed that in both the models no cerebrospinal fluid leak was detected when compared with the sutured control group. On histological examination of the dura, two months post-treatment, fibrous tissue connection was present between the dura and the implanted fascia in the TA/PF gel group in comparison to the sutured control group. To assess the antioxidant effect of the gel, a ROS detection assay was performed. Increased malondialdehyde levels and decreased Superoxide dismutase levels indicated increased ROS production at the injury site which was reversed within 7 days of implanting the gel. The gel also reduced apoptosis of local neural cells and regulated inflammation by inhibiting microglial activation and immune cell infiltration in comparison to the control group. It was also observed that a compact scar barrier was formed along the border of the lesion to restrain the spreading of inflammation and maintain neurons viability and promote neurological functional recovery.

Qi et al. [191] synthesized film constituting graphene oxide (GO) and L-theanine (TH) with poly(lactic-co-glycolic acid) (PLGA). To evaluate the properties of each component, neural stem cells were cultured on glass, PLGA, PLGA/GO, and PLGA/GO/TH films. After H_2_O_2_ treatment, it was observed that there was a significantly higher survival rate of cells in the PLGA/GO/TH group than in other groups. Cell proliferation and differentiation were highest in PLGA/GO/TH group when compared with other groups at 7 days. It was concluded that TH increased the expression of anti-apoptotic protein Bcl-2 and decrease the level of caspase-3 by inhibition of extracellular-signal-regulated kinase (ERK) and c-Jun N-terminal Kinase (JNK) pathways inhibiting the reactive oxygen species production.

## 6. Conclusions and the Future Outlook

Oxidative stress is one of the major causes of neurodegenerative diseases which can be effectively reduced with the use of an appropriate antioxidant. In order to develop the endogenous defense against oxidative stress, quite a few therapeutic approaches have been suggested. A large panel of natural antioxidants exhibit a protective effect in preventing cellular oxidative stress, but their low bioavailability and inability to cross the BBB restrict their therapeutic activity at the targeted injury site [192,193]. Over the past decades, noteworthy achievements were made to develop novel nanotechnology-based systems for therapeutic delivery of natural antioxidants to enhance antioxidant bioavailability and clinical efficacy for the therapy and prevention of neurodegenerative diseases [192,194]. Nanotechnological approaches (in particular quantum dots) can be employed to penetrate the BBB due to their small size and deliver antioxidants at high enough concentrations to mitigate oxidative stress in neurotrauma and neurodegenerative diseases [195,196]. The use of artificial antioxidant systems such as bioinspired catalytic nanoparticles, nanoscale drug delivery systems loaded with enzymes, and other nanomaterials, were proven to be some effective strategies [197]. These systems have delivered many advantages such as superior tissue targeting, extended half-life, enhanced solubility and stability, enriched epithelium permeability and bioavailability and reduced side effects. Additional benefits of antioxidant nanotherapies are intrinsic ROS-scavenging properties, stable anti-oxidative activity, and remarkable pharmacokinetics [198]. Despite the promising results, antioxidant concentration (and dosage) should be carefully considered due to the potential “boomerang effect” and the role of endogenous free radicals in various biochemical signaling pathways necessary for normal functioning [199,200]. However, there has been a lack of a comprehensive review that covers all the current and recent advances in these antioxidant bioengineering strategies except for a few which are confined to neurodegenerative diseases but not for neurotrauma and neuroregeneration [192,197]. Therefore, in this review we complied various antioxidants that were demonstrated to be effective against neurological disorders and neurotrauma either by direct administration or by incorporating them within bioengineered scaffolds.

Direct administration of antioxidants is commonly observed, however, it demonstrated disadvantages such as low availability, poor absorption rare, less BBB penetration, local tissue irritability, etc. Whereas the bioengineering strategies discussed have shown localized and efficient delivery, biocompatibility, sustained release and kinetics. It was also observed that in most of the studies, the efficacy of the treatment and recovery was much improved when a combinatorial strategy was employed, i.e., by amalgamating natural/synthetic antioxidants with the biomaterials in various forms. Nerve guidance conduits incorporating various antioxidants were studied extensively in vitro and in vivo but carefully controlled clinical trials have to be explored further. Though most of the reported studies showed successful in vitro and pre-clinical results, they have not led to clinical practice yet due to a lack of evidence about degradation products and associated long-term neurotoxicity [198]. Additionally, in vitro studies were limited to 2D cultures, which cannot recapitulate the innate in vivo environment. Therefore, studies assessing antioxidant properties on 3D cultures and the development of 3D organoid models through technologies such as 3D bioprinting would help to emulate the native environment in a better way. Additionally, the use of 3D in vitro disease models could be superior alternative strategies for pre-clinical animal models which were reported to have inconsistent results and ethical concerns [197]. Newer methodologies such as 3D bioprinting technology could be employed to augment the existing bioengineering-based antioxidant therapies specifically for neuro regenerative applications. Neurons, mainly in the CNS, do not regenerate very properly due to the inherent inhibitory environment and secondary damage caused by oxidative stress. Hence tissue engineering approaches focused on providing a favorable environment, by mimicking the ECM milieu, stimulating the intrinsic cells to repair, and restoring the functions. Additionally, the radical scavenging ability with potential antioxidant-based bioengineered scaffolds would ameliorate functional neuronal recovery. Three-dimensional bioprinting provides very good control over the design and microstructure of such scaffold and this makes it a very potent and attractive technology to fabricate complex neural scaffolds, along with cells and antioxidants embedded together. Thus, such advanced technologies combined with traditional approaches could pave the way for faster relief of neurotrauma as well as promote the neuroregeneration and recovery in neurological diseases of PNS and CNS.
